# Clinical feasibility and safety of third space robotic and endoscopic cooperative surgery for gastric gastrointestinal stromal tumors dissection: A new surgical technique for treating gastric GISTs

**DOI:** 10.1007/s00464-019-07223-w

**Published:** 2019-10-17

**Authors:** Feiyu Shi, Yingchao Li, Yanglin Pan, Qi Sun, Guanghui Wang, Tianyu Yu, Chengxin Shi, Yaguang Li, Hongping Xia, Junjun She

**Affiliations:** 1 Department of General Surgery, The First Affiliated Hospital of Xi’an Jiao Tong University, No. 277, Yanta West Road, Xi’an, 710061 Shaanxi China; 2 Department of Gastroenterology, The First Affiliated Hospital of Xi’an Jiaotong University, No. 277, Yanta West Road, Xi’an, 710061 Shaanxi China; 3 Xijing Hospital of Digestive Diseases, Fourth Military Medical University, Xi’an, 710032 Shaanxi China; 4 Laboratory of Cancer Genomics, National Cancer Centre, Singapore, 169610 Singapore

**Keywords:** Gastrointestinal stromal tumors, Gastric submucosal tumors, The third space, Endoscopic technique, Submucosal injection, Robotic surgery

## Abstract

**Background:**

Surgical management of gastric gastrointestinal stromal tumors (GISTs) has evolved towards minimal invasiveness. Laparoscopic wedge resection and laparoscopic and endoscopic cooperative surgery had been considered as standard surgical treatments for gastric GISTs > 2 cm. However, stomach deformation and the full-thickness gastric defect caused by these procedures may increase the risk of morbidity. To address these problems, we developed a novel technique, third space robotic and endoscopic cooperative surgery (TS-RECS), which could dissect the tumor entirely while preserving the intact mucosal layer. Here we performed a prospective evaluation of the feasibility and safety of TS-RECS.

**Methods:**

Patients with gastric GISTs were recruited between April 2018 and April 2019. During the operation, the gastric GIST was located by endoscopic view firstly and the submucosal injection was performed. The tumor was then dissected through robotic surgery. Clinicopathological characteristics, operative data, adverse events, and follow-ups were prospectively collected and analyzed.

**Results:**

A total of 20 patients with gastric GISTs received TS-RECS. The mean tumor size was 33.0 ± 7.3 mm. R0 resection was achieved in all patients with a median operation time of 115 min and a median blood loss of 20 ml. The integrity of mucosal layer was maintained in 95% (19/20) of the patients. All patients started oral diet on postoperative day 1 or 2, staying in the hospital for a median of 6 days after surgery. There were no major adverse events. Local or distant recurrences were not observed during a median follow-up period of 10 months.

**Conclusions:**

Our study suggests that TS-RECS appears to be a feasible and safe technique which could be an alternative method for resecting gastric GISTs > 2 cm.

**Clinical Trials:**

ClinicalTrials.gov NCT03804762.

**Electronic supplementary material:**

The online version of this article (10.1007/s00464-019-07223-w) contains supplementary material, which is available to authorized users.

Gastrointestinal stromal tumors (GISTs) are the most common submucosal tumors (SMTs) in the stomach [1]. Surgical resection is recommended for lesions > 2 cm in many guidelines because of the malignant potential of the tumors [2–4]. Due to the rare possibility of lymphatic metastasis of GISTs, local R0 resection with less extensive excision is always the target of surgical treatment [2–4].

In recent years, surgical management of gastric GISTs has evolved towards minimal invasiveness, such as laparoscopic wedge resection (LWR), laparoscopic and endoscopic cooperative surgery (LECS). LECS was first reported by Hiki et al. in 2008 [5]. Many studies had demonstrated that LECS is a safe, feasible, and effective procedure for treating gastric GISTs [6–9], especially advantageous in removing the tumor completely without excessive resection of the gastric wall. In recent years, LECS has been considered as a standard treatment for gastric GISTs in Japan [10]. However, there are also several limitations in the LECS procedure. For instance, the full-thickness incisions caused by LECS may lead to contamination of the abdominal cavity during the operation and thereby increasing the risk of postoperative intra-abdominal infections or GI leakage [11–13]. Furthermore, some technical limitations in laparoscopic equipment, such as the inflexible devices, unstable movements, and unsatisfying 2-D views, compared with Robotic surgery may increase the difficulty during the tumor dissection and the risk of postoperative morbidity [14].

In recent years, there were increasing studies about the application of robotic surgery in gastric cancer [15–17], and the clinical advantages of robotic gastrectomy had been initially confirmed. However, the use of robot surgery in gastric GISTs was rarely reported. In this study, we presented a new surgical procedure for gastric GIST dissection, which combined the endoscopic technique and robotic surgery, and termed third space robotic and endoscopic cooperative surgery (TS-RECS). With the help of the third space established by endoscopic techniques, we can dissect gastric GISTs entirely while preserving the integrity of the mucosal layer during the dissection of tumor by using robot surgery, which could address those above problems relating to LECS or laparoscopic surgery. Between January 2018 and April 2018, we completed several TS-RECSs to treat patients with gastric GISTs, with favorable short-term results. Here, we conducted a prospective study to assess the feasibility and safety of TS-RECS for gastric GISTs.

## Materials and methods

### Patients and study design

After the study was approved by the Institutional Review Boards of the First Affiliated Hospital of Xi’an Jiao Tong University (XJTU1AF2018LSK-168), patients with gastric GISTs were prospectively recruited from April 2018 to April 2019 and received TS-RECS at our institution. The inclusion criteria included the following: (1) patients with gastric GISTs originating from muscularis propria (MP) layer diagnosed by endoscopic ultrasonography (EUS); (2) a maximal transverse diameter of the tumor ranging between 20 and 50 mm; and (3) no evidence of tumor metastasis on preoperative evaluations. The exclusion criteria included the following: (1) patients with serious systemic comorbidities, such as severe heart failure, respiratory failure or uncontrolled hypertension; (2) patients with advanced malignant tumors; (3) emergency operations due to complete intestinal obstruction, perforation and hemorrhage caused by the tumor; (4) patients with ulcers related to the tumor; (5) patients with contraindications for general anesthesia; and (6) pregnancy. Before the procedure, all patients signed an informed consent. Preoperative CT and EUS were performed in all patients. This study was registered in ClinicalTrials.gov (number NCT03804762).

### TS-RECS procedure

**Fig. 1:**
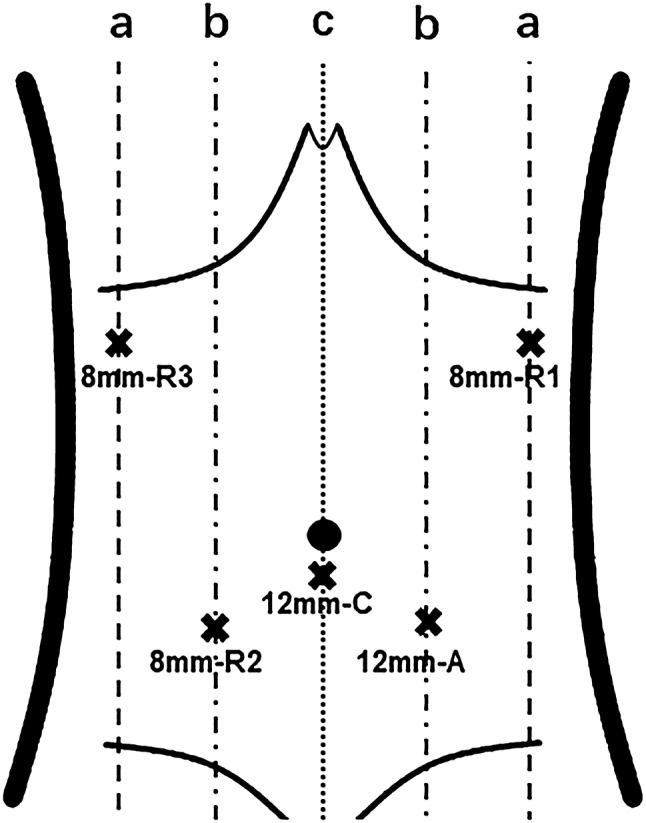
The trocar placement of TS-RECS: R1,2,3, robotic arm 1,2,3; C, robotic camera; A, assistant; a, anterior axillary line; b, midclavicular line; c, linea mediana ventralis

**Fig. 2:**
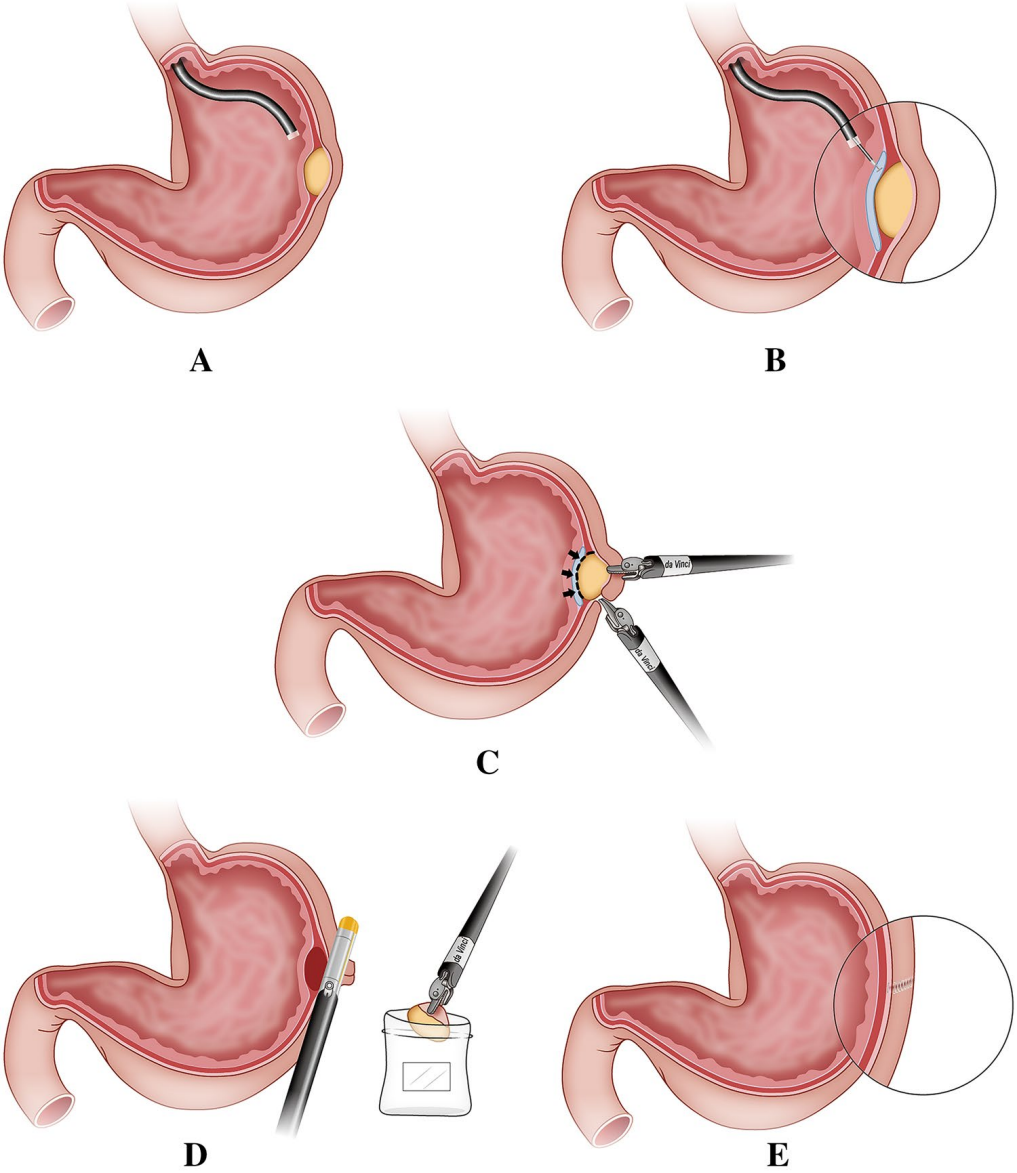
Main steps of TS-RECS for dissecting gastric GISTs. **A** Confirmation of the tumor location. **B** Establishment of the third space through submucosal injection. **C** Dissection of the tumor around the black line by da Vinci robot. **D** Closure of the seromuscular incision and collection of the excised tumor in specimen bag. **E** Intact mucosal layer and minimal deformation in stomach after the tumor dissection by TS-RECS technique

**Fig. 3:**
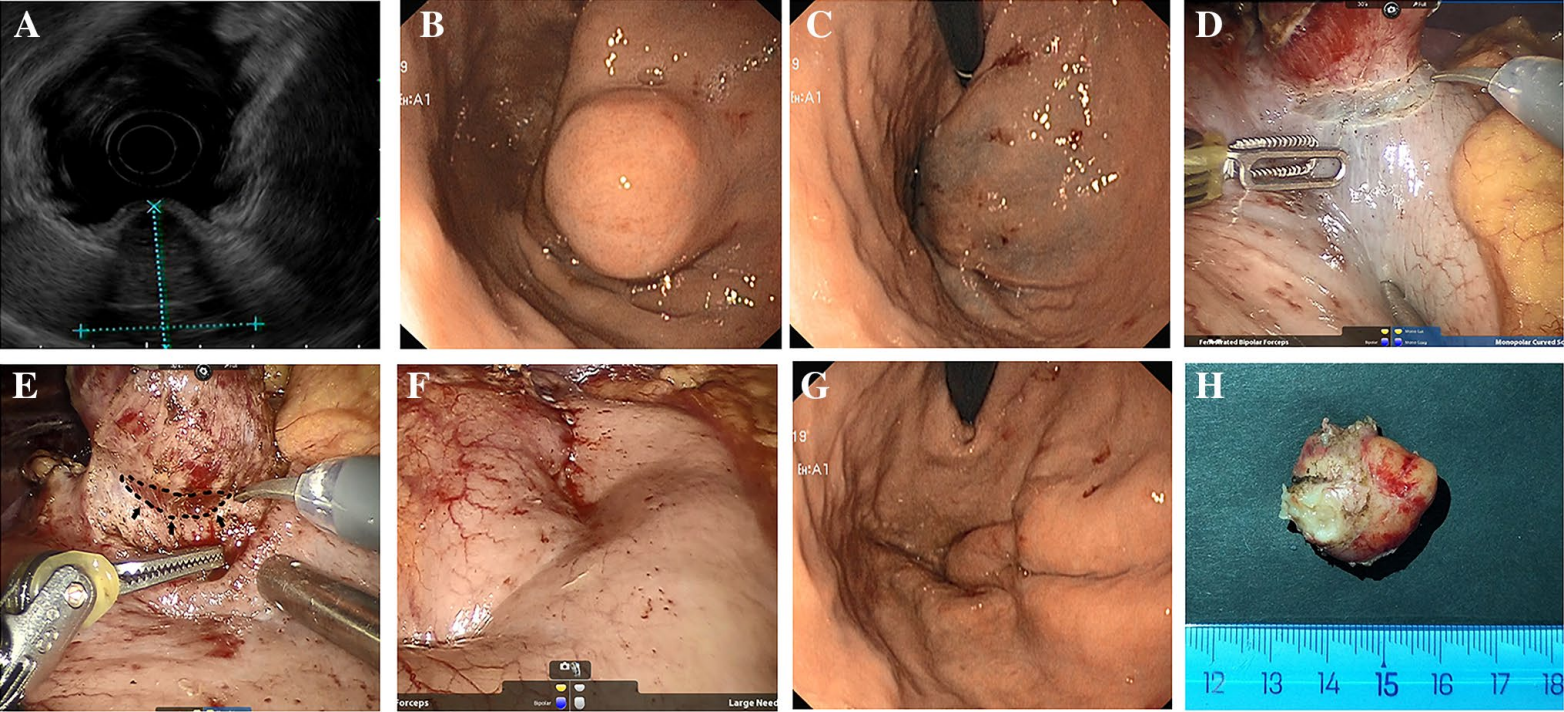
Dissection of a gastric GIST by TS-RECS. **A** The GIST originated from MP layer on EUS. **B** The Gastric GIST on endoscopic view. **C** After the establishment of the third space by endoscopic submucosal injection around the tumor. **D** The seromuscular incision at the edge of the lesion. **E** The dissection of the tumor by robot surgery (black line and arrow indicates the third space liquid cushion). **F** The seromuscular incision in robotic view after the tumor dissection using TS-RECS. **G** The intact mucosal layer in endoscopic view after the tumor dissection using TS-RECS. **H** The resected spacemen

TS-RECS was performed by an experienced surgeon (J.J.S.) and an experienced endoscopist (Y.C.L.). All procedures were performed using the da Vinci Surgical System (Intuitive Surgical, Sunnyvale, CA) and a single-channeled endoscope (GIF-Q260 J; Olympus). The procedural details included the following six steps (Video Fig. 1-2 and Figs. 1, 2 and 3 described all TS-RECS details):

#### (i) Setting up the da Vinci surgical system

The patient was placed in the reverse Trendelenburg Position and was put under general anesthesia. The da Vinci camera port was inserted via a 12-mm trocar below the umbilicus. A pneumoperitoneum was established, maintaining an intra-abdominal pressure between 10 mmHg and 12 mmHg. Four operational ports (one 12-mm port and three 8-mm ports) were inserted into the left upper, left lower, right upper, and right lower quadrants of the abdomen, respectively, using camera visualization (Fig. 1). The location of the 12-mm port was determined based on the location of the tumor. The spaces between adjacent trocars were kept > 8 cm to effectively reduce adjacent robotic-arm interference.

#### (ii) Confirmation of gastric GISTs locations

The endoscopist stood on the left side of the patient and confirmed the tumor’s location by endoscopic image (Figs. 2A and 3B). When the tumor had a predominant intraluminal growth pattern, a mucosal bulge would be identified. If a tumor had a predominant extraluminal growth pattern which may not present a mucosal bulge, robotic view and preoperative CT imaging would help to localize the tumor under endoscopic view.

#### (iii) Local blood vessel preparation and division of adhesions (if necessary)

According to the location of the tumor, we divided and dissected the omentum and blood vessels around the operation area using ultrasonic shears. We avoided unnecessary local preparation and division of adhesions, minimizing manipulations in the areas of blood vessels.

#### (iv) Establishment of the third space by submucosal injection

We chose 4–5 sites around the tumor for endoscopic submucosal injection (Figs. 2B and 3C). A mixed solution (10% glycerol fructose, 4% methylene blue) was injected submucosally into the periphery of the tumor to open up the third space and mark the tumor location. After the submucosal injection, the potential space between the mucosal layer and the GISTs would be enlarged by the expanded submucosal layer, and the tumor location was marked blue.

#### (v) Robotic submucosal dissection of the tumor

We started the tumor dissection by making a seromuscular incision at the edge of the lesion (Fig. 3D), and then precisely dissected the connective tissue surrounding the tumor in the third space (submucosal space) using monopolar curved scissors until the tumor was entirely removed from the gastric wall (Figs. 2C and 3E).

#### (vi) Closure of the seromuscular incision

With the dissection finished, the excised specimen was collected intracorporeally into a prepared bag. The seromuscular incision in the gastric wall was closed using robotic hand-suturing technique or stapling device (Figs. 2D and 3F). We then checked the closure with the endoscopic air-leak test and ensured that there was no bleeding from endoscopic and robotic imaging. The specimen bag was retrieved via the extended infra-umbilical trocar incision.

### Perioperative management

Prophylactic administration of antibiotics (2 g Cefmetazole) was intravenously injected half an hour before the operation. In terms of postoperative diet, a small amount of water was given by mouth 12 h after operation. When patients were asymptomatic or tolerable, a liquid diet was started. During the follow-up period, all patients underwent EUS and abdominal CT every 3 to 6 months to evaluate whether there were residual or recurrent tumors.

### Data collection and outcomes definitions

We prospectively collected clinicopathological data, including age, sex, BMI, preoperative symptoms, tumor location, tumor size, and histopathological diagnosis. Operation-related characteristics included operation time, estimated blood loss, ratio of intact mucosal layer, R0 resection, complications rates, time to oral diet, length of postoperative hospital stay, hospitalization expenses, and evidence of residual or recurrent tumors on follow-up.

The primary outcome were incidence of adverse events, which included intraoperative complications (hemorrhage, injury to visceral organs and vessels, and anesthesia complications), and postoperative complications (infections, intra-abdominal/intraluminal bleeding, gastric stasis and leakage) graded according to the Clavien–Dindo classification [18]. Secondary outcomes included rate of R0 resection, ratio of intact mucosal layer, operation time, estimated blood loss, time to oral diet, and length of postoperative hospital stay. R0 resection was defined as the complete en bloc resection with microscopically negative margin. Operation time was defined from the time of docking to the time of trocar incision closure.

### Statistical analysis

Statistical analysis was performed using GraphPad Prism version 6.0 (GraphPad Software, La Jolla, CA, USA). The results were shown as the mean ± SD (normally distributed) or median plus range (non-normally distributed).

## Result

### Clinicopathologic characteristics

The clinicopathologic characteristics are summarized in Table 1. This study included 20 patients (13 females and 7 males) with a mean age 54.5 ± 10.7 year and a mean BMI of 22.3 ± 1.7 kg/m^2^. The main complaints from patients included epigastric pain (*n *= 9), epigastric distention, or discomfort (*n *= 6). Tumors were located in the upper third portion of the stomach in 9 patients, middle third in 7, pyloric region in 2, and esophagogastric junction in 2. The mean tumor size was 33.0 ± 7.3 mm. Pathologic diagnosis was confirmed by immunohistochemistry, and the results showed GISTs in 20 patients, including 19 low risk and 1 intermediate risk according to the Fletcher classification. On discharge, we recommended imatinib treatments for the GIST patient with intermediate risk.

**Table 1:** Clinicopathologic characteristics of patients treated with TS-RECS

Number of patents	*N *= 20
Sex, male; female, *n*(%)	7 (35%); 13 (65%)
Age (years)	
Mean ± SD; (range)	54.5 ± 10.7; (37–80)
Body mass index (kg/m^2^)	
Mean ± SD; (range)	22.3 ± 1.7; (19.5–25.2)
Symptoms	
Epigastric pain, *n*(%)	9 (45%)
Epigastric distention, *n*(%)	3 (15%)
Epigastric discomfort, *n*(%)	3 (15%)
No symptom	5 (25%)
Tumor location	
Esophagogastric junction	2 (10%)
Upper third (anterior/posterior/lesser/greater)	4/1/0/4 (20%/5%/0%/20%)
Middle third (anterior/posterior/lesser/greater)	1/3/1/2 (5%/15%/5%/10%)
Low third (anterior/posterior/lesser/greater)	0/0/0/0 (0%)
Pyloric	2 (10%)
Tumor size (mm)	
Mean ± SD; (range)	33.0 ± 7.3; (24–50)
Pathological diagnosis	
GIST(low risk), *n*(%)	19 (95%)
GIST (intermediate risk), *n* (%)	1 (5%)

### Operation-related outcomes and adverse events

Operation-related data are showed in Table 2. R0 resections were achieved in all patients (20/20) and the integrity of the mucosal layer was maintained in 95% (19/20) of the patients. The median operation time for TS-RECS was 115 (90–160) min with an estimated median blood loss of 20 (5–100) ml. There was no postoperative leakage, intra-abdominal infections, or gastrointestinal function-related complications. In terms of other adverse events, one patient (F/80, GIST) experienced postoperative pneumonia (Clavien-Dindo grade II) and received intravenous antibiotics treatment. The patient recovered uneventfully and was discharged on postoperative day 10. All patients started oral diet on postoperative day 1 or 2, staying in the hospital for a median of 6 (4–10) days after surgery. During a median follow-up period of 10 (3–15) months, no local or distant recurrences were found in all patients.

**Table 2:** Operation-related outcomes, expense, and follow-up of patients treated with TS-RECS

Outcomes *N *= 20	
Operation time (minutes)	
Median; (range)	115; (90–160)
Estimated blood loss(ml)	
Median; (range)	20; (5–100)
Number of R0 resection, *n*(%)	20 (100%)
Number of Integrity of mucosal layer, *n*(%)	19 (95%)
Adverse events	
Pneumonia (Clavien-Dindo Grade II)	1 (5%)
Time to oral diet (days)	
Median; (range)	1; (1–2)
Postoperative hospital stay (days)	
Median; (range)	6; (4–10)
Hospitalization expense (US dollars)	
Median;(range)	7793.25; (7128.8–11880.7)
Follow-up time (months)	
Median; (range)	10; (3–15)

## Discussion

Due to the malignant potential of gastric GISTs, surgical resections are considered the first-line treatment for the lesions > 2.0 cm [2, 3]. For decades, the efforts to explore the optimal management of gastric GISTs > 2 cm had never stopped, during which the third space and the da Vinci robot had gained more attention. In our study, we report the use of TS-RECS, a technique that combined endoscopic techniques, the third space and robotic surgery, demonstrating that it was a feasible and safe procedure to dissect gastric GISTs while preserving the integrity of the gastric mucosa.

The third space is actually a potential space, located between the muscularis mucosa (MM) and the muscularis propria (MP) layer, hence also termed as submucosal space [19, 20]. The submucosal space had been described as a valuable operation space for the diagnosis and treatment of upper gastrointestinal submucosal tumors (SMTs) [21], especially for the SMTs originating from the MP layer [22, 23]. Most gastric GISTs originates from the MP layer and are covered by normal mucosa [1]. After the submucosal space was created and expanded by the submucosally-injected liquid cushion, the layered structure between mucosa and tumor would become more distinct, and the tumor location as well as the resection region can then be marked in robotic view. In this way, it was possible to dissect the lesion precisely without causing damage to the mucosal layer. Moreover, for the lesions nearby the esophagogastric junction or pyloric region, the technique can dissect the tumor completely and avoid a total/proximal gastrectomy. In our study, R0 resection was achieved in all patients (including 2 in esophagogastric junction and 2 in pyloric region), and there was no patient need to convert to open surgery or proximal gastrectomy. The integrity of the mucosal layer was maintained in 95% (19/20) patients. One patient (M/58, GIST) required a full-thickness resection during the operation, due to the firm adhesion between the tumor and the mucosal layer. Thus, we had to resect part of gastric mucosa in order to ensure the complete resection of the tumor. In this case, an Endo-GIA was used to close the full-thickness incision.

The intact mucosal layer could serve as a protective barrier to peritoneal soiling [24], and the risk of intra-abdominal infection may theoretically be decreased by changing the traditional class II surgery (Clean-contaminated) into a class I surgery (Clean) [25]. In addition, TS-RECS technique minimized the surgical invasiveness and maximally preserved the gastric volume as well as anatomical and functional integrity, which can help achieve a lower risk of infection-related and gastrointestinal function-related complications. Although these complications were found in 4–4.8% of patients in two previous large LECS study [8, 10], these complications were not observed in our study, supporting the hypothesis that the integrity of mucosal layer may prevent complications relating to the full-thickness resection. Although other modified LECS procedures such as combination of laparoscopic and endoscopic approaches to neoplasia with a non-exposure technique (CLEAN-NET) [26], non-exposed endoscopic wall-inversion surgery (NEWS) [12], and closed LECS [13] also reduced complications relating to the full-thickness resection, they were technically demanding and time-consuming.

In the past several years, the surgical strategy for stomach neoplasm had evolved towards minimally invasive techniques. The feasibility, safety, and efficiency of da Vinci robot in stomach surgery had been demonstrated in many retrospective and prospective studies [15–17, 27]. In the present study, TS-RECS took advantage of the superiority of da Vinci robot to improve the efficiency and precision of the procedure, which facilitated precise anatomical dissections of the tumor layer by layer. Compared with previous reports on LECS [8, 10], the operation-related results of TS-RECS showed reasonable blood loss and postoperative recovery, acceptable complication incidence and seemed to have a decreased operation time (LECS: 174–190 min/TS-RECS:115 min). There are several possible reasons for the decreased operation time. One is the difference in the details of dissection of tumor between two procedures. LECS technique is completed by endoscopic submucosal dissection and then laparoscopic seromuscular dissection, which is meticulous and time-consuming. However, in TS-RECS technique, all dissection procedures are completed by robotic surgery alone because the technical advantages of da Vinci Surgical System (e.g., flexible and precise multi-joint forceps, tremor-filtering function and a 3-D high-quality view) enabled us to perform each meticulous procedures more efficiently [17]. In addition, other possible reasons for the shorter operation time are that most of tumors in our study are located at the anterior wall and the greater curvature, which were easier to resect compared to lesions in the posterior wall and the lesser curvature.

In terms of oncological safety, first we limited the use of TS-RECS in gastric GISTs or other gastric SMTs originating from MP layer with the intact capsule because if the tumor has mucosal defect or ulceration, tumor cells might scatter to the abdominal cavity, resulting in peritoneal metastasis. Second, the ruptured tumor may lead to peritoneal seeding, thus the most important principle during surgery is to maintain the integrity of the tumor capsule, which is technically demanding during tumor dissection. However, the technical advantages of robotic surgery helped us to find the proper layer and avoided injuring the tumor capsule during tumor dissection. In this study, the intact tumor capsule was confirmed macroscopically and microscopically in all patients. Third, as mentioned before, TS-RECS dissected circumferentially the tumor without damaging the mucosal layer, which suggested the tumor enucleation with minimal margins. Although the resection margins in this study were smaller than recommendations, the R0 resection with negative margin was confirmed pathologically in all cases. In addition, some studies had reported the favorable oncological safety and prognosis of tumor dissection with minimal margins. Joo et al. compared long-term oncological outcomes of endoscopic circumferential resection and surgical resection for upper GI GIST, and results suggested there is no significant difference in recurrence rate between the two groups (2.2% and 5.0%; *p *= 0.586) during 45.5 months of follow-ups [28]. Chen et al. also reported a favorable oncological outcomes of tumor enucleation for 2.0–5.0 cm upper GI SMTs, including GISTs, which showed 0/177 recurrence rate during the 36-month median follow-ups [23].

It should be noted that this present study had several limitations. First, this was an initial pilot study with a small cohort of 20 patients. Because the operation fees for robotic surgery were not covered by the Chinese Health Insurance, the patients have to pay an additional admission fee (around US$3000) when they undergo robotic surgery. Thus, it was hard to recruit sufficient cases for the study with a large sample size during the 12-month study period. Second, the 10-month median follow-ups was insufficient to support the efficacy from an oncological standpoint. Undoubtedly, a larger-scale studies with long-term follow-up are needed in the future to further validate our findings. Nevertheless, considering that this is the first prospective study to report the use of robotic and endoscopic cooperative surgery for the resection of gastric GISTs while preserving the intact mucosal layer, we believe that this study represented a significant initial experience in the combination of endoscopic submucosal space and robotic surgery. With the help of robotic techniques, surgical management will be more precise and less invasive. Second, we limited the procedure in selected patients as mentioned above, which made the special technique has a narrow indication. But, with the development of precision medicine in surgery, surgical techniques should be tailored to patient’s and tumor’s characteristics, helping the patient obtain the biggest benefit from surgery.

In conclusion, our preliminary data showed TS-RECS might be an alternative method for treating gastric GISTs > 2 cm, having the potential advantages of higher efficiency and fewer adverse events.

## Electronic supplementary material

Below is the link to the electronic supplementary material.

Supplementary material 1 (MP4 44928 kb)
